# RubiN – continuous care in regional networks: a study protocol for a prospective controlled trial

**DOI:** 10.1186/s12877-021-02106-z

**Published:** 2021-03-16

**Authors:** Simone Gloystein, Friederike Thomé, Katja Goetz, Nicole Warkentin, Karola Mergenthal, Fabian Engler, Volker Amelung, Matthias Arnold, Felix Freigang, Ann-Kathrin Klähn, Sonja Laag, Neeltje van den Berg

**Affiliations:** 1 Institute for Community Medicine, Section Epidemiology of Health Care and Community Health, University Medicine Greifswald, Greifswald, Germany; 2 Institute of Family Medicine, University Medical Center Schleswig-Holstein (UKSH), Lübeck Campus, Lübeck, Germany; 3 Institute of General Practice, Goethe-University, Frankfurt am Main, Germany; 4 Private Institute for Applied Care Research, inav Berlin, Berlin, Germany; 5 Department for Product Strategy/ Development, BARMER Health Insurance, Wuppertal, Germany

**Keywords:** Geriatric care, Interprofessional cooperation, Regional networking, Regional health care, Interdisciplinary, Health services research, Case management, Geriatric assessment, Age

## Abstract

**Background:**

The health care situation of geriatric patients is often multifaceted, complex and often overlaps with social living conditions. Due to the lack of cross-sectoral and interprofessional health care geriatric patients often, receive insufficient care. Only a holistic view enables a comprehensive evaluation of the complex health risks, but also the potential to preserve the health of geriatric patients. The implementation of cross-sectoral, multi-professional case management could reduce the gaps in care, improve the autonomy of the geriatric patients in their own homes, and allow them to retain it as long as possible.

The “RubiN” project examines the effects of multi-professional, cross-sectoral and assessment-based case management on the quality of the care of geriatric patients. The results of the study aim to show whether geriatric patients receive better care using case management than patients who receive standard health care. In addition, data on the effects of case management on practices of general practitioners (GP), the satisfaction with the care concept amongst the case managers, patients and relatives will be collected. Furthermore, a health economic analysis will be carried out.

**Methods:**

The project is designed as a prospective controlled study and compares geriatric patients from practice networks in different regions in Germany. Inclusion criteria are: Age ≥ 70 years and care requirements from two different care complexes (identified with the screening instrument ‘Angelina’-questionnaire). The intervention is the use of a geriatric case management, where health care is organised based on patient-specific care requirements. Five practice networks of physicians will implement the intervention (*n* = 3200 patients) and three practice networks will serve as the control group (*n* = 1200 patients). The primary endpoint is the ability to manage activities of daily living, measured using the Barthel Index. The patients in the intervention group receive geriatric case management and the patients in the control networks receive standard care (“care as usual”). The analysis of the primary data, which is pseudonymised, occurs according to the intention-to-treat principle. For this purpose, the endpoints will be analysed using a group comparison after 12 months. For the health economic analysis, secondary data from the statutory health insurance providers will be included in the analysis, in addition to the primary data. Data for the analysis of the effects the concept has on the GP practices as well as on the satisfaction of the project participants will be collected with questionnaires and interviews with experts.

**Discussion:**

The implementation of cross-sectoral and interdisciplinary geriatric case management has been a topic of discussion for years, whereby positive effects have already been-shown. This planned study will be the first evaluation of the effect of case management for geriatric patients with a very large sample. In addition, the effects of case management on the GP practices and also on the relatives of the geriatric patients will be shown. It is intended that the study results pave the way for a widespread implementation of this concept.

**Trial registration:**

German Clinical Trials Register, ID: DRKS00016642. Registered on 29 October 2019 - Retrospectively registered.

## Background

The combination of a low birth rate and the high life expectancy of the cohort with the highest population among the intermediate age groups today (baby boomers = those born in the years from 1959 to 1968 [1]) is leading to major changes in the age structure of the German population. The number of elderly people is set to rise considerably. In 2013 there were already 4.4 million people aged 80 and over in Germany, which represented 5% of the population at the time. Their number will increase 40% by 2030 reaching 6.1 million, and in the year 2060 there will be in total approx. 9 million, which is about double the figure from 2013 [2]. Simultaneously, there will be a change in the prevalence of illnesses in Germany. For example, in comparison to 2007, a 20% increase in the prevalence of diabetes mellitus or a 41% increase in the prevalence of visual impairments in the German population is to be expected by 2050 [3].

These changes in the age structure of the population and in the prevalence of illnesses in Germany will have serious implications in terms of increased patient numbers, in particular with regard to age-related chronic illnesses and multimorbidity [4, 5]. A geriatric patient with multiple illnesses is often simultaneously affected by acute and chronic illnesses as well as functional limitations and is often at risk of being unable to remain in his/her own home.

The health care system in Germany, which to date has been highly sectored, and focused on physicians and acute inpatient care, does not meet the requirements of geriatric patients who need cross-sectoral care, also across professions, with a focus on the general practitioner (GP) [6].

### Case management for geriatric patients

Case management, which originated in the Anglo-Saxon countries, has also been introduced to and further developed in Germany since the 1990s. So far, there have been several projects dealing with case management based on the conditions in Germany [7]. The Advisory Council on the Assessment of Developments in the Health Care Sector (SVR) recommended the involvement of non-medical staff in the care of patients as early as 2007 [8].

In the AGnES project (community-based, e-health-assisted systemic support for primary care), for instance, it was shown that specially qualified caregivers and medical assistants can carry out tasks delegated to them by the GP to a high quality standard. This laid the foundation for the possibility to delegate tasks in GP practices, which has been possible since 2009 within the framework of standard care [9].

However, the implementation of case management in the German health care system is still slowly progressing, although e.g. the coordination and accordingly close monitoring of care are particularly important. The need for case management also arises from the increasing complexity of care of geriatric patients in various life situations and the complexity of the care structures in the German health care system. It is of particular importance that case management occurs across sectors and professions and not, as has been the case to date, health care is predominantly separated into outpatient and inpatient care and often uncoordinated. Case management’ goal is to guide each individual patient through the health care system, which often involves resolving complex issues and requires a high degree of coordination. This includes establishing contact with relevant service areas (e.g. social, health and insurance) and to steer efficiently the process of the provision of services. Thus, it involves continuous monitoring over a longer period of time and beyond the confines of the individual service areas.

The aim of case management is to ensure that the spectrum of tasks is carried out in close cooperation with the GP (ranging from the case intake, case assessment, goal and assistance planning, implementation and review of the help plan through to evaluation) [7]. Furthermore, it assures that the care situation is coordinated and that the retention or improvement of the independence of the geriatric patients in their own homes can be achieved.

Case management within the RubiN framework is not limited anymore to individual service providers. Instead, the individual service providers (GP practices) can access a joint pool of specially trained case managers within their network of physicians. In other words, the case managers are employed in the network of physicians and use a constantly expanding regional network, e.g. consisting of local facilities, care support points, social services, rehabilitation centres, physiotherapists, occupational therapists, support groups, nutrition counselling services, sports clubs, etc..

### Aims of the project

The main question of the RubiN project is whether multi-professional, cross-sectoral and assessment-based case management within the framework of a network of physicians improves the state of health of geriatric patients and their care situation in the network.

This question is to be examined on several levels (patient and relatives/ close personal contacts, service providers, practice networks) with different methods and evaluation endpoints.

The primary patient-level question is whether case management leads to patients in the intervention group being better able to manage activities of daily living after 12 months than those in the control group (primary endpoint: Barthel Index [10]).

At the level of relatives and close personal contacts, the objective is to determine whether the use of case management in the intervention group eases the situation and leads to greater satisfaction with regard to the care of geriatric family members.

The comparison between intervention group and control group in terms of the service providers is concerned with the effects of the intervention (case management). This includes the time taken between the identification of a care requirement and it being resolved, changes in terms of issues at the interface between different service providers, the satisfaction with the care situation and the quality of the teamwork, which is necessary due to the delegation of services.

At the level of the practice networks, the study explores whether the case managers’ curriculum corresponds with fulfilling tasks in the different networks. These tasks comprise whether diagnostic and care services can be assigned to the respective service providers (GP, specialist, case manager) based on a defined algorithm. It includes identifying barriers or beneficial factors due to networking of practices, or which proportion of the identified care services was able to be implemented after 12 months and by whom (GP, specialist, case manager, nursing services, etc.).

## Methods/ design

### Study design and procedure

The RubiN project is to be conducted as a prospective controlled intervention study in a clinical setting. Five practice networks, which are certified according to § 87b SGB V implement regionally adapted assessment-based case management. This certification of the practice networks provides a verification of special management skills [11]. The patients of the GP practices in these practice networks make up the intervention group. There are 640 patients per network, thus 3200 patients in total. Three further practice networks conduct the care of the patients as usual, without implementing case management. The patients of the GP practices in these three practice networks form the control group. It is comprised of 400 patients per network, thus 1200 patients in total (see Sample size calculation).

All patients receive the baseline assessment (BL) (see Table 1). In the intervention networks the patients included in the study then receive geriatric case management, designed according to patient-specific care requirements, for a period of 12 months. The patients in the control networks receive “care as usual”.

**Table 1:** Outcome parameters for the levels (patients and relatives and close personal contacts)

Outcome	Measurement instrument/ operationalisation	Data collection point
**(A) Patient level**		
**(1) primary**		
Activities of daily living	Barthel Index	BL; FU12; FU21
**(2) secondary**		
Health situation with regard to geriatric aspects	ANGELINA screening	BL; FU12
Managing necessary daily tasks in the household	Instrumental activities of daily living (IADL)	BL; FU12
Mobility	Timed Up and Go, Tinetti’s mobility test	BL; FU12
Cognitive Abilities	DemTect	BL; FU12
Falls	Fall protocol	when/if a fall occurs
Quality of life	WHOQOL-OLD/ WHOQOL-BREF	BL; FU12
Malnutrition	Minimal Nutritional Assessment, MNA-Elderly	BL; FU12
Sociodemographic variables (age, gender, family background, further relevant sociodemographic data)	own design	BL; FU12; FU21
**(B) Level of relatives and close personal contacts**		
Easing of the burden	Burden Scale for Family Caregivers	BL; FU12
Sociodemographic variables (age, gender, family background, further relevant sociodemographic data)	own design	BL; FU12

*BL* baseline, *FU12* follow-up after 12 months, *FU21* follow-up after 21 months

**Fig. 1:**
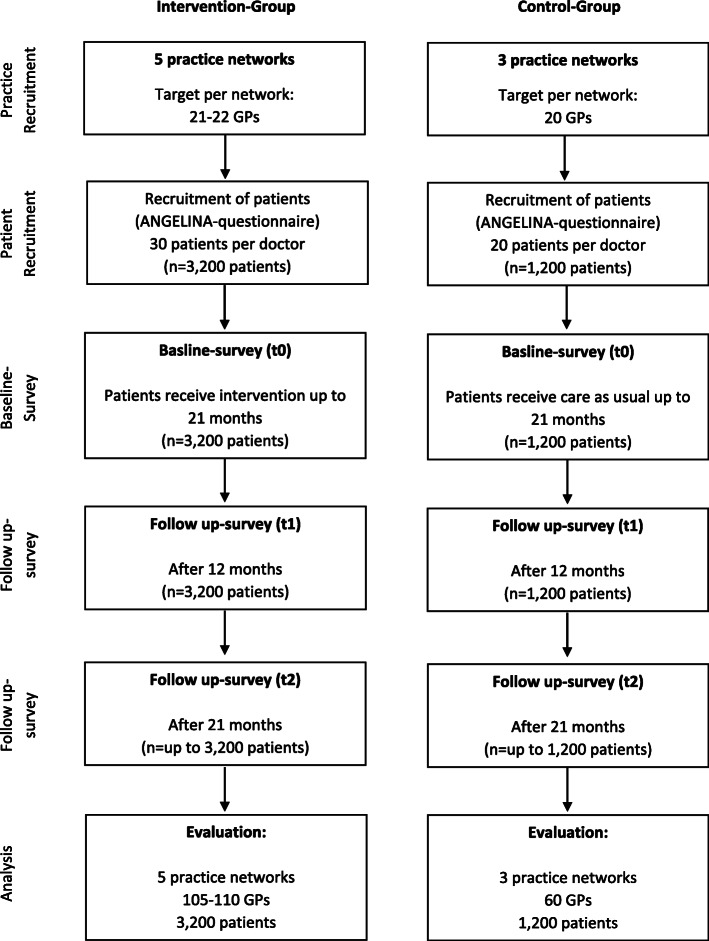
Project implementation plan

After 12 months, an assessment is then carried out again, with all patients: the 12-month follow-up (FU12). For all patients who reach 21 months of participation within the duration of the project there is an additional collection of data on the primary endpoint (Barthel Index) (the 21-month follow-up (FU21)) (see Fig. 1).

### Identification of eligible patients

In the GP practices that are participating in the practice networks a short questionnaire covering the most important geriatric topic areas will be completed for all patients who are aged 70 or over (e.g. regarding housing, assistance needs, medication, mobility, senses) (Angelina questionnaire [12]). If patients have assistance needs from two different topic areas they are eligible for the study and will be asked to participate in the project.

### Project participation, inclusion and exclusion criteria

#### Inclusion and exclusion criteria for participating practices

Inclusion criteria: The physician is working in a practice as an specialist for internal medicine, GP or specialist for general medicine and must be a member of the respective network of physicians (certified according to § 87b SGB V).

Exclusion criterion: GPs who work exclusively in a private practice.

#### Inclusion and exclusion criteria for participating patients

Inclusion criteria: geriatric patients: patients ≥70 years old with at least two geriatric feature complexes (i.e. ≥2 points in the ANGELINA-questionnaire from at least two different topic areas), a sufficient command of the German language.

Exclusion criteria: patients who are living in inpatient care facilities at the time of recruitment, patients with a terminal illness and/or using specialised palliative care (specialised palliative outpatient care, a hospice), patients with bipolar disorder or other severe psychiatric illnesses.

### Intervention

Health care professionals (qualified nurses, therapists or health care assistants (HCA)) receive the multiprofessional geriatric care training “GeriNurse” [13]. This is a 210-h training course (online and practical) and contains the following topics: health and project management, risk identification (preparation of clinical pathways), case management, risk management, accounting procedures, controlling, care management (regional neighbourhood management, public health, committee work).

Four case managers per practice network will receive the qualification, thus 20 in total for the RubiN project.

Based on the results of the patient’s baseline assessment the trained case managers perform a risk assessment. Based on the identified care requirements, patients are categorised as mild, moderate or severe cases. This provides the structure to determine the workload for the case managers in each case for the following 12 months (see Table 2) and a patient-specific, optimal treatment and/or care plan for the geriatric patient is prepared. The case managers monitor developments and progressions on a case-by-case basis, i.e. they make assessments and determine whether the care plan is to be followed or if it needs to be corrected or adjusted. The case managers identify care gaps and if required assess the care situation of these patients by means of case discussions and “round table” conferences. In doing so, they work closely with the GP and coordinate the care of the geriatric patients by being active across sectors and working with all other health professions in their respective practice network.

**Table 2:**
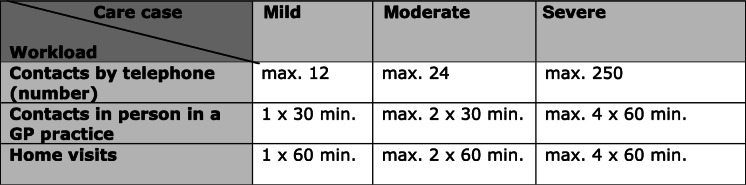
Care-related workload per year for each patient, according to case severity

In addition, the case managers establish a network specifically for geriatric care in the regions, with appropriate communication and collaboration structures.

### Baseline assessment

The baseline assessment (BL) consists of measurement instruments which collect multidimensional data on the different aspects of geriatrics and the patient-relevant endpoints (see Table 1).

### Follow-up assessments

After 12 months, the 12-month follow-up assessment (FU12) takes place both in the intervention group and the control group. It uses the same measurement instruments as the baseline assessment. After a further 9 months, this is followed by the 21-month follow-up assessment (FU21) for the patients who reach this point within the duration of the project. Aside from the personal data of the patient only data related to the primary endpoint, the Barthel Index, is collected again.

### Sample size calculation

The number of cases was estimated for the primary endpoint (Barthel Index) of the RubiN project. The following parameter assumptions were made:

- Error 1st type (α): 5%.
- Error 2nd type (β): 15% (≙ power of 85%)
- Effect size (d): 0.3
- Intervention/control ratio (on the patient level): 3/1
- Assumed intracluster correlation coefficient: 0.15
- Assumed drop-out rate: 33%

To demonstrate an effect of d = 0.3 with the mentioned assumptions, *n* = 534 participants are needed (400 in the intervention group and 134 in the control group). It is estimated that about 22 practices will participate in each practice network, each of which will be able to recruit an average of 30 participants. With an assumed intracluster correlation coefficient [14] of 0.15, a “design effect” of 5.5 for the regional design must be taken into account in the sample size planning. This increases the necessary sample size to *n* = 2937.

With an assumed dropout rate of 33%, 4384 (2937/0.67 = 4384) participants should be included, rounded up to 4400. The 5 intervention networks will each recruit about 640 participants (3200), the 3 control networks will each recruit about 400 participants (1200) (ratio 2.7:1, rounded up 3:1).

### Data collection

#### Level of the patients and relatives or close personal contacts

At the level of the patients and their relatives or close personal contacts primary and secondary data are collected and analysed.

The primary data of the patients enrolled in the study and of their relatives or close personal contacts are collected by practice employees who are trained for this task (control networks) and case managers (intervention networks) by means of the assessments created for this purpose (baseline and follow-ups). The primary data is recorded in the data management system CentraXX (Kairos GmbH, Bochum) at the Institute for Community Medicine, University Medicine Greifswald using the eCRFs (electronic case report forms) completed based on the assessments.

Within the framework of the process evaluation, the study also investigates the acceptance of the intervention. Focusing on the perspective of patients and their relatives and close personal contacts, as well as its feasibility and implementation, it is evaluated by means of case analyses, focus group meetings or expert interviews. Appropriate instruments have been developed for this purpose.

#### The level of the practices and networks of physicians

At the level of the practices and networks of physicians, surveys and process analyses will also be conducted.

In order to conduct the process evaluation, alongside the quantitative and administrative data from patients, relatives and close personal contacts, medical and non-medical service providers, additional data and information will be generated by means of guided expert interviews and the formation of interprofessional focus groups.

For the evaluation of the special features of practice networks with regard to the course of the health care provision in terms of interprofessional cooperation and the special role of case management in this scenario a special interview technique will be employed: the Pictor technique [15, 16].

### Health economics

The health economic assessment focuses on two research objectives: 1) The project’s impact on overcoming the sectoral divide as experienced by health providers and patients. 2) The project’s economic impact measured as the cost per reduction in ambulatory sensitive hospital cases (ACSC), which is interpreted as a quality indicator for ambulatory services [17].

The health economic analysis includes quantitative and qualitative components using primary patient and service provider information and the secondary data from statutory health insurances. Cost include all relevant claims experienced by the health insurances, e.g. of outpatient, inpatient and emergency treatments, of medicinal products, remedies, aids and rehabilitation (if these are paid for by the health insurance provider).

ACSC are measured as the difference in the average frequency in the intervention and control groups.

The analysis of the sectoral gaps uses qualitative methods. Based on semi-structured expert interviews with medical and non-medical professions across the different practice networks, information is gained about experiences with sectoral barriers in the provision of care.

The goal is to highlight how the RubiN care concept can contribute towards solving the issues identified in relation to interfaces in health care provision.

### Ethics and data protection

All of the recruitment and data collection procedures within the framework of the study occur on the basis of the ethical principles for medical research involving human subjects (The Declaration of Helsinki) as well as the recommendations for safeguarding good scientific practice of the German Research Foundation (DFG), the Guidelines and recommendations for ensuring Good Epidemiological Practice (GEP) [18], the principles of the “Good Practice in Secondary Data Analysis” [19], the Memorandum III “Methods for Health Services Research” [20] and also the standards of the German Evaluation Society (DeGEval) [21].

There is a harmonised data protection concept for the RubiN project for all partners in the consortium.

The project has received the approval of the Ethics Committee of the University of Greifswald (BB188/18), issued 17.01.2019.

### Data analysis

#### Patient level

To test the structural equality of the intervention and control groups the characteristics of the patients and the GP practices at the point in time t_0_ (baseline) are examined using descriptive and analytical statistical procedures (group comparisons).

The primary analysis of results follows the intention-to-treat principle and includes all the patients involved in the study. After the follow-up (t_1_, 12 months) the patients from the intervention and control group are analysed with regard to the different outcomes in a group comparison. The evaluation is performed with multivariate models and multilevel analyses, in order to be able to adjust for possible differences between the service providers and between the practice networks. The analyses are conducted using the statistics software SAS 9.4.

#### Level of the practices and networks of physicians

The qualitative data collected are digitally recorded and subsequently orthographically transcribed and assessed by means of content analysis [22] with an appropriate software (i.e. MAXQDA 2020).

The quantitative data are evaluated descriptively using the statistics programme SPSS 25.0. In order to ensure a multi-perspective view of the research topic, the interpretation of the results of the qualitative and quantitative analyses occurs in a heterogeneously composed research group [23]. The objective is a triangulation of the collected qualitative and quantitative data [24, 25]. The main focus is on the qualitative analysis.

#### Health economy

Within the framework of the health economic evaluation the cost-effectiveness of the care concept is examined. For this purpose, there is firstly an analysis of the utilisation of health care services and the associated costs and secondly, there is an assessment of the frequency of avoidable hospitalisations.

In order to be able to make statements about the cost-effectiveness, the billing data of the respective health insurance providers will be merged with the outcome variables collected in the project in a trust centre. By comparing the gains in effectiveness and additional expenditure versus the economic efficiency of standard care, the RubiN care concept can be evaluated.

## Discussion

The implementation of geriatric case management across sectors and professions has been discussed for years. Often there are financial barriers, which prevent it from being implemented. Concepts have already been developed and realised in GP care. Examples are medical assistants who use GP-based case management to assist in the care of e.g. depressive patients [26], patients with arthritis [27], chronically ill patients [28] and also geriatric patients [9]. This can result in significant differences in the care of the patients. The use of case management versus “care as usual” currently depends on the respective standards in the individual practices, their interpretation of good geriatric care, as well as their situation in terms of finances and staffing.

The current geriatric care is illustrated in this study by the care of the patients in the control group and the study will show how “care as usual” is interpreted within certified networks of doctors. The comparison is a deciding factor as to whether geriatric case management has an effect on patient care and whether it can be considered as positive.

The intention of this study is to show whether, compared to geriatric patients receiving the previous standard care in practice networks, geriatric patients who have experienced care with case management within certified practice networks without gaps between care levels and sectoral boundaries receive more optimal care and support. Furthermore, we are investigating whether GP practices experience a reduction in the workload associated with the care of geriatric patients due to the implementation of case management. The RubiN project also investigates how the introduction of a geriatric case management impacts relatives and the health care situation. It is hoped that this will provide important insights for the later transfer of the care concept to standard care.

### Limitations

This project constitutes a regional comparison. This is statistically not as robust as a grouping by randomisation at the patient level (RCT). An RCT was not possible here as the intervention is implemented as a regional structure.

### Strengths

One of the strengths of the project is the large number of patients included. Furthermore, the project is implemented in real care. Due to the few criteria excluding patients from participation in the study, the patients represent the actual health care situation. The results are thus readily transferable to other regions and patients. Moreover, the analyses take place at various levels (patients, relatives and close personal contacts, practices, networks of doctors, economy), so that a comprehensive overall picture emerges.

## Data Availability

The final trial dataset will be accessible to the study investigators. Data may be also avalaible for project-related research questions in coordination with the project consortium. With a reasonable request, contact the corresponding author SG.

## Abbreviations

ACSC: Ambulatory Sensitive Hospital Cases
AGnES: Community-Based, e-Health-Assisted Systemic Support for Primary Care
BL: Baseline
DeGEval: German Evaluation Society
DFG: German Research Foundation
eCRF: Electronic Case Report Form
FU12: 12-Month Follow-Up
FU21: 21-Month Follow-Up
GEP: Good Epidemiological Practice
GP: General Practitioner
HCA: Health Care Assistant
SGB: German Social Insurance Code
